# Critical success factors for the implementation and adoption of e-learning for junior health care workers in Dadaab refugee camp Kenya

**DOI:** 10.1186/s12960-019-0435-8

**Published:** 2019-12-09

**Authors:** Aude D. Burkardt, Nicerine Krause, Minerva C. Rivas Velarde

**Affiliations:** 1 Faculty of Medicine, University of Geneva, 4 rue Gabrielle-Perret-Gentil, CH-1211, 14 Genève, Switzerland; 2 Department of Radiology and Medical Informatics, HI5lab (Health Informatics for Innovation, Integration, Implementation and Impact) Faculty of Medicine, University of Geneva, CMU/1 rue Michel Servet, 1211 Genève 4, CP Switzerland

## Abstract

**Abstract:**

This paper presents the results of a case study that analyses the critical factors that influence the implementation of professional health education via blended learning in Dadaab refugee camp. It explores innovative solutions to the issues facing refugees looking for professional health training, namely the health workforce shortage and lack of training opportunities. It outlines social and political factors that impact professional health education for refugee youth. It outlines barriers and facilitators on the implementation of ‘Distance Basic Training of Healthcare Professionals’, a blended training course provided by the University of Geneva to junior health care personnel in Dadaab Refugee camp.

**Methods:**

This case uses mixed methods. Descriptive statistics drawn from online surveys, learning analytics data, and exchanges on online forums and student chat groups are all used. Qualitative methods consist of two focus groups, comprising of all students (*n* = 27) were convened, as well as, individual semi-structured interviews with 14 of the 27 students; three with managers from the health service who supervised enrolled students; and two with senior managers who were responsible for staff and training decision-making. Qualitative data was transcribed, and thematic analyses were applied.

**Results:**

The results demonstrate that barriers for the implementation of professional education in a refugee camp emerged not only from the constraints on the environment, but also from barriers stemming from legislation and administrative procedures. Data suggested weaknesses on the education system could be addressed by providing students with extra-curricular support, information and communications technology (ICT) literacy, and promoting mechanisms for peer support while broadening entry requirements to increase the enrolment of female students. Finally, providing internationally credentialed courses and transferable skills enables professional pathways for refugee students.

**Discussion:**

Blended learning enables the design and delivery of high-quality medical education that is sustainable and relevant in a particular environment, e.g. refugee camps. Furthermore, the research reveals that building education pathways could enhance numbers of health workers with the appropriate skillset to serve communities.

## Introduction

Since the beginning of the Somali Civil War, there has been a drastic reduction of qualified medical staff, doctors, nurses and technicians and a destruction of training institutions and universities in Somalia [1]. Non-governmental organisations (NGOs) and United Nations agencies allow the training of most of the staff in the refugee camp. This paper presents a case study based on the implementation of ‘Distance Basic Training of Healthcare Professionals’, a blended training course provided by the University of Geneva (UNIGE). The course complements on-the-job training of junior health personnel in Dadaab. Data from this case study contributes to building knowledge on how technology, through blended learning, could enable capacity building amongst the health workforce in refugee camps. This paper concludes with recommendations that will benefit policymakers and educators involved capacity building on human resources for health in fragile contexts.

### Dadaab—26 years later

Dadaab refugee camp was constructed in 1992. It was originally intended to temporarily house refugees fleeing the civil war in Somalia. It is now home to approximately 238 794 refugees and asylum-seekers; approximately, 96% of the refugees come from Somalia [1]. The complex comprises four camps: Hagadera, Dagahaley, Ifo and Ifo 2 camps. In 2013, the governments of Somalia and Kenya along with the United Nations High Commissioner for Refugees (UNHCR) signed the Tripartite Agreement designed to promote the voluntary return of Dadaab’s refugees to Somalia [2].

In May 2016, the Kenyan government announced the immediate closure of Dadaab and repatriation of its 350 000 Somali refugees [3]. This decision was later overturned by the Kenyan High Court, which ruled that the repatriation decision violated the 1951 Refugee Convention [4]. Despite the court ruling, the future of Dadaab remains uncertain. A permanent encampment policy, which mandates refugees remain in the designated refugee camp, is in place.Refugees do not have the right to work, and they face severely restricted opportunities in terms of training and higher education [5].

Recently, education skills development has become increasingly important in formulating solutions for refugees. The Nairobi Declaration on Durable Solutions for Somali Refugees (2017) has called on states to enhance education, training and skills development for refugees [6]. This will enable refugees to contribute to host communities and the reconstruction of Somalia. Therefore, it is important to investigate how these opportunities occur. Empirical evidence is needed to inform responses in terms of education and health. Innovative education programmes and partnerships that provide medical and paramedical training bridging the deficit of medical care in Somalia and employment and education opportunities can be facilitated on their return to the camp [7].

### Professional health education and Dadaab

While improvements have been made in the provision of primary and secondary education, access to higher education is deteriorating for refugees [8]. Fewer than 1% of refugees worldwide have access to higher education [8]. Looking for innovative learning methods that respond to and address the realities of refugee youth is very pertinent and important.

Existing literature shows that e-learning has advantages in low-resource settings. In the African context, it has increasingly been used in health care to support the delivery of learning in outcome-based-education schemes in low-resource contexts [9, 10]. There are various e-learning formats, either as text-led webpages, blended learning programmes, massive open online courses (MOOCs), or online clinical simulations. These approaches remove barriers and enlarge the training capacity for health science education in Africa [11–13]. Innovative solutions have tackled infrastructural limitations through low-bandwidth distance technology [14–16]. Furthermore, significant progress has been achieved in transforming health professional education by enhancing south-to-south partnerships and resource sharing using e-learning [11]. Nonetheless, literature providing evidence on innovative solutions for young refugees to access professional health education is practically absent. This paper addresses this gap.

The few studies identified focused on technical inputs and what effect providing this type of education to refugees could have for population health. For example, two empirical studies on the Thailand-Burmese border were identified: the work of Minden [17], who explored the impact of training midwives, and Turner et al. [18], who evaluated the outcomes of providing refugees with training regarding supportive care for babies in a neonatal intensive care unit. Furthermore, Chen et al. [19] analysed the impact of delivering sexual and reproductive training to lay refugees in 48 camps in Guinea. Their results showed a positive impact on the control of STDs and maternal health interventions. In the Americas, Cropley [20] utilised a controlled post-test, community-based study to evaluate the effects of a health education intervention that involved training up lay refugee health workers to deliver education on child malaria treatment-seeking practices amongst rural refugee mothers. Their results also showed a positive effect on treatment-seeking behaviours. The overall upcoming trend of positive health outcomes associated with training refugees in healthcare. Ehiri et al. [21] studied the impact of basic health service training for lay refugees and internally displaced populations and demonstrated that such training raised positive outcomes in a population’s health. Ehiri et al.’s results reiterated the importance of professional health education and further opportunities for refugees.

Moving towards professional training in 2017, Mbai et al. [22] reported on the absence of professional health education in the Dadaab refugee camp. They investigated the training priorities of young refugees and the priorities for service providers. The authors claimed that community health workers were perceived as the primary health providers. Their research showed that training in areas such as nursing or medicine was not an option given the limited health infrastructure in the Dadaab camp. Their work led to the creation of a Bachelor of Science in Community Health Education, in partnership with Moi University, Kenya.

A year later, Rivas Velarde [23] reported that several training programmes offering professional health education were occurring in Dadaab. The author listed the online degree Borderless Higher Education for Refugees (BHER) and Moi University, as well as others offered by the Windle Trust Kenya & World University Services of Canada, Kenyatta University Campus Dadaab, The Lutheran World Federation and North Coast Medical Training College. Rivas Velarde noted that it was not possible to determine the reach or the level of success of such initiatives given the lack of public information available or scientific validation of these academic approaches. This lack of information on training and education as well as research exploring the impact of such training raised questions about the lack of consideration of refugee youth as agents of change for health systems; furthermore, not documenting experiences, successes and lesson learned impact negatively on the planning of future initiatives and the use of limited funds available in the region.

## Methods

This case study utilised mixed methods combining quantitative analysis with descriptive statistics of students’ academic progress and participation in online platforms and qualitative interviews and focus groups to understand the contexts and experiences of students and key personnel involved in supervising some of the student’s work performance.

Descriptive statistics were drawn from online surveys, learning analytics data, and exchanges on online forums and student chat groups (see Additional file 1 ‘results of profile correlation’). These approaches evaluated student academic progress, utilisation of ICT platforms and participation in academic discussions. Additionally, two focus groups, comprising of all students, were convened, and semi-structured interviews were conducted with 14 of the 27 students. The remaining 13 students were given the chance to answer the same set of questions via online questionnaires. Half of them answered the questionnaire. Other collected data included five semi-structured interviews, three with managers from the health service who supervised enrolled students and two with senior managers who were responsible for staff and training decision-making. Qualitative data was transcribed, and thematic analysis was applied, working inductively with the transcripts. Additionally, field notes and grey literature from a fact-finding mission involved informal information sessions and meetings with several key Kenyan and Somalian personnel from the health and education sectors. Careful and repeated reading of the dataset led to themes being identified by senior author and grouped into categories through discussion with other authors. Data was compared and contrasted with quantitative data.

### Course characteristics

This research focused on an 8-month blended learning course entitled ‘Distance Basic Training of Healthcare Professionals’ offered in Dadaab, from February 2017 to February 2018. The original length was prolonged from 8 to 12 months. A student hub was equipped with desktop computers and high-speed internet access and was located at IFO1-Dadaab for students. Transport to the site was subsidised by course funds.

Course materials were delivered using a Moodle platform. A Universal Serial Bus (USB) stick provided to all students contained all written materials, bypassing internet connectivity issues. Course materials were complemented by teaching support from medical students, via e-tutorials on WhatsApp. Students also received a face-to-face teaching input.

Two student cohorts successfully completed the course. Of the 27 students initially recruited, 18 finished the course and 14 successfully passed the final exam. All students admitted were Somali refugees and high school graduates. The gender balance for graduates was five women and 13 men. The majority were active in the health workforce or had held health-related ‘incentive’ posts in the past.^1^ An international NGO acting as the implementing partner in the field supported this course, as well as a university in Somalia that explored the possibility of having the students continue their studies in Mogadishu, which resulted in 2 students using their computer lab to complete their course in Mogadishu.

### Ethics

The research protocol was submitted to the Swiss Ethics Committees on research involving humans. Participation was voluntary, and confidentiality and anonymity were assured. Written consent was obtained from participants. The committee had no objections to the ethical aspects of our study proposal, and it was cleared for approval. The assigned protocol number was 2017-00632.

## Results

The results outline some critical factors that impacted the implementation of this blended learning course. Four interrelated themes that outlined social, technological, and political factors that impacted the delivery of the course emerged, namely (i) contextually sensitive learning for refugees, (ii) extra-curricular student support, (iii) the use of digital tools and (iv) professional pathways available to refugee students. They will be now outlined:

- Contextually sensitive learning for refugees

This theme compiled data on contextual and infrastructural barriers impacting on students and programme management. Participant narratives recounted how the camp’s highly volatile environment, interspersed with unresolved educational weaknesses, made it very difficult to enter let alone succeed in higher education.

- Everyday life in Dadaab

Dadaab is geographically and socially isolated, insecure, and governed by extremely strict safety protocols. These factors impacted the delivery and planning of the course. Logistics, such as distributing course materials, were challenged by long waiting periods and unconventional transportation routes and relied on the individual’s determination and schedules rather than structured mechanisms such as functional postal services.

Furthermore, the layout of the camp and threats of violence imposed significant delays to students’ progress. Sometimes, camp security operations obliged students to stay in designated shelters and miss study sessions for weeks at a time. Additionally, on various journeys to the hub, students were detained by the police, interrogated, and asked for identification cards, which they did not have. The police also required approval for commutes between sites. Participants reported feeling insecure and intimidated. Furthermore, during the rainy season, participants were unable to reach the student hub due to heavy floods. The following quotes represent these general feelings;

There are tough operation going on site, nobody cannot go outside we have postponed our trip to the hub^2^. (1)

Security is hard, we get stopped many time, (name/title of a local key actor) helped us, the police detained us because we do not have ID. And they never knew us or sometimes there are no vehicles coming to IFO*.* (4)

b) Enhancing female participation

Security threats and cultural barriers also emerged, for example, difficulty in engaging with female learners. After the initial admission call, a second call was issued targeting only prospective female students. This measure allowed the course to attain 30% female representation. Females were granted admission with lower grades when compared to some male students. Despite this measure, gender had no influence on the marking and results of the exams. The overall score of both cohorts is 37.4% for females and 42% for males (*p* value 0.40). See further details on academic performance in Additional file 1. Since the two highest scores were in cohort 2, the isolated scores of cohort 1 were 40.6% for females and 37% for males

Female students recalled seeking approval from their families to engage in education or employment. They were pressured to get married and have children, instead of pursuing higher education. Decision-makers and supervisors at the hub were enthusiastic about having more female graduates engage in professional health education. They all described positive changes in female participants, such as better communication skills and students being more outspoken and confident. They also showed greater motivation to learn. Some managers pointed out that

These female graduates had become role models, not only to junior staff at the health sector in Dadaab, but also to their community at large. Other managers recalled staffing issues from having to free their staff to go for training. But there were more of those who celebrated having training available and were optimistic about managing potential shortages. The following statements elaborate on this:

Women will marry and they will leave school. But now is a new world I want to learn. (3)

She motivating other youth, other women to study. This training gives them respect in the community. (B)

c) Legal frameworks

Barriers stemmed from legislation and administrative procedures. Although Kenyan legislation does not prevent refugees from entering professional education, the encampment policy, along with challenges related to weak borders between Kenya and Somalia, had a negative impact on refugee youth participation in higher education.

A partnership with a Somali institution was tested. Students were given the option to continue their course at a university if they travelled to Somalia. Students reported that having access to professional health education that was not bound to a single location for the entire duration of the 8-month course was key for refugee youth in Dadaab. All students were doubtful about their future whereabouts, because they felt that Dadaab was closing or they were considering repatriation to Somalia or settlement at a third location. They reported that online learning meant they could follow their course work, even with the uncertainties that marked their lives. The following quote captured this impression:

Having classmates in Somalia taught me a lot, it taught me that UNIGE is not only here ... If you move you are not left behind, wherever I go I can get access to my studies without any limitations. (7)

Understanding the context where learning occurs requires going beyond security constraints, scarcity, and social and structural barriers. The following theme presents findings related to how learning styles and education gaps played major roles in the rolling out of professional health education in Dadaab.

ii) Academic and extra-curricular support

Unresolved educational weaknesses at Dadaab negatively impacted students taking this course. Data suggested that secondary education models in Dadaab were not sufficiently preparing students with critical thinking skills which are necessary for higher education. Furthermore, the data showed that education gaps tended to be significant; therefore, extra-curricular support was essential for the implementation of the programme. These findings are illustrated below.

- Educational gaps and peer support

During the admissions process, the administrative team became aware that most students had been out of the educational system for more than 5 years. This suggested challenges, as students would need extra support to re-enter education after such a long gap. Furthermore, this transition would be exacerbated as they were entering a flexible, self-regulated learning environment. Students conveyed that re-entering education was a challenge and that managing the online learning platforms was intimidating at first, but they all enjoyed the flexibility that online learning provided. They also stated that peer-to-peer support in computer literacy skills was key to successfully following the course. They entered into study routines and overcame fears and doubts about their learning progress. Setting up group study sessions also was key in promoting peer-to-peer support and easing students back into education. The following quotes illustrate this:

We meet up for the first time at the hub, some learnt how to access the platform... and together we succeeded. (FG)

b) Course design

Barriers to learning emerged from the course design. Module 1, which covered key principles of biology, anatomy, and physiology, was found to be too dense and too much to be covered in the allocated time, whereas module 2 which was more practical and oriented towards the acquisition of knowledge on frequent and/or relevant medical conditions in Eastern Africa was mostly found adequate and was generally more successful. Therefore, using more accessible learning content with slides highlighting the key elements would be more adequate. The results show the amount of contents and the learning schedule must be adapted to be reachable, while still maintaining an expectation level compatible with tertiary education programmes accredited by the University of Geneva.

c) Barriers from the perspective of students

**Fig. 1:**
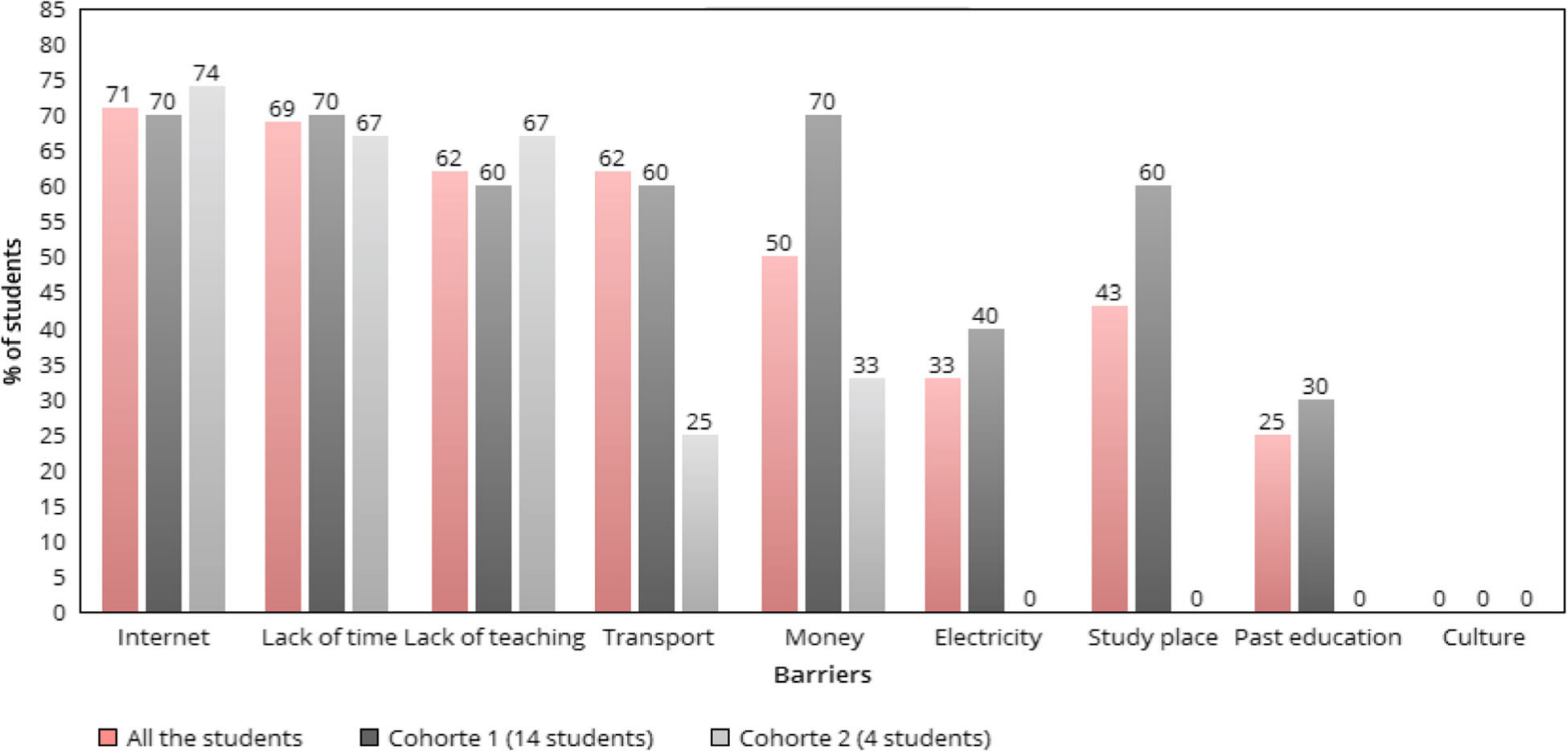
Main obstacles ranking from the student’s perspective

**Fig. 2:**
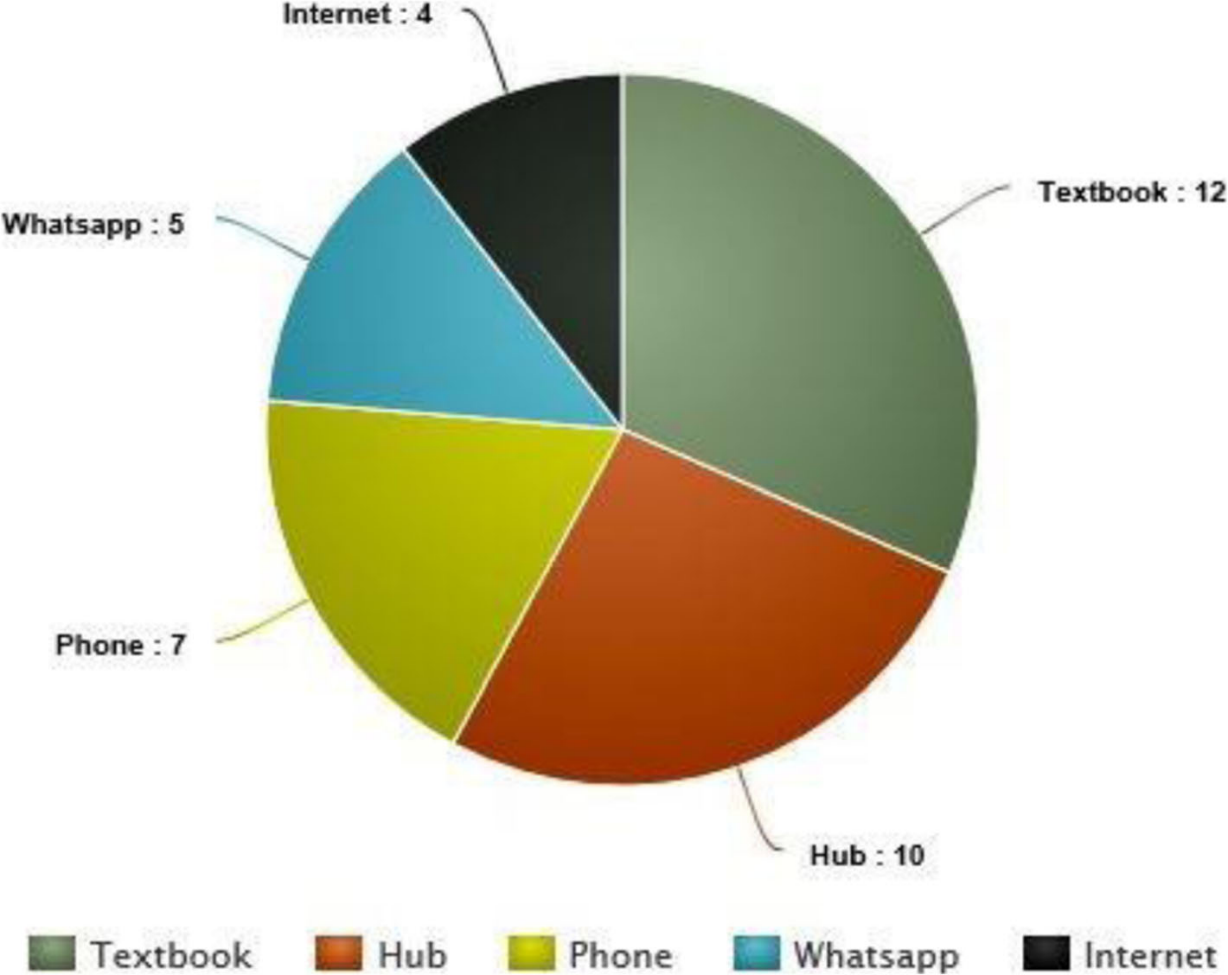
Main sources used by the students in both cohorts

Furthermore, other obstacles were explored. Figure 1 ranks obstacles drawn from student’s experiences. The views from the first cohort and the second cohort of students are shown separately, demonstrating that no factors have a statistically significant impact on scores according to the *p* value, except for the motivation level (nor the gender, the sources studied, etc.) (Figure 2 Main Sources used by the students in both cohorts).

Perhaps, a deep assessment of student’s expectations of the programme and their professional wishes could need to be given a stronger consideration onto the selection criteria for this programme and similar initiatives carried out in similar context.

d) Higher education skills

Fostering deep learning was a much weightier issue. Students were insufficiently prepared to embrace critical thinking. They valued memorising the course material instead of understanding it. Their narratives suggested that in previous learning they had adopted more passive roles and memorising was expected. The following quotes show this:

(In our previous learning) We have a teacher and someone help us to memorised the class. (FG)

(Suggesting improvements on the course) It will be good to have a teacher to help us memorising the book. Sometimes... you cannot always count with your classmates. (FG)

The face-to-face unit adopted a problem-based learning approach, and results were evaluated by oral exam. This unit demanded significant skills of the lead facilitator. Results suggested that more is needed to be done to develop critical thinking skills for students in Dadaab. This passive learning attitude was a barrier and was much more difficult to tackle. It was a physical hindrance to learning. E-mentors found it very difficult to engage with students in a meaningful give-and-take discussion during tutorials; they tried different ways to engage with students; personalised tutorials were initially tried; however, it was more successful to run guided group tutorials using videos and enhancing prompt and immediate communication. Analytical data from the WhatsApp group indicated no links between high participation and better grades; thus, this communication method was celebrated amongst students as it enhanced interactions amongst students, e-mentors, and administrators. Aside from e-tutorials, online quizzes were conducted and successfully completed.

Using a mobile phone app for learning was new to students, as was for many the use of online learning platforms such as Moodle. The following themes present a more detailed analysis of participant views and the use of technology for learning

iii) Use of digital tools

Access to blended learning allowed participants to build on their technical capacity and contribute to the professional health workforce in Dadaab. It pivoted the development of ICT skills, enabling participants to access other online learning platforms and resources, as well as connecting to labour markets in Dadaab and abroad. This section of the report shows the potential of mobile devices for learning in isolated areas like Dadaab.

- ICT to facilitate learning

The course utilised stand-alone computers at the learning hub and apps on smartphones. Participants said that attending the learning hub was very important, but they reported that Internet connectivity was a constant issue. In contrast, smartphones were perceived as more efficient and they covered a greater range of functions. They were logistically used for organising group trips to the hub, to alert participants of abnormal camp activities and security threats, and to access course material by using USB On-The-Go (OTG-B) adaptors for their USB sticks. Not only did participants cite mobile devices as highly advantageous, but managers also expressed interest in exploring how mobile devices could be used to provide continuing education to their staff and themselves. The following quote reflects this: *‘*At first people were not sure about this type of training (online). Now people have seen that it works and they are interested, (student) was the genie in the bottle. I would be interested in training using my phone or other accessible format.’ (A)

Although ICT skills were low at first, WhatsApp proved to be very popular with participants in Dadaab. They all used it regularly and were familiar with its features, such as voice and video features and web versions. Almost all participants owned a smartphone, only two participants borrowed smartphones to access the App. A key feature of the App was its low data requirements. However, accessibility was not the only advantage. The platform enhanced cooperation and peer-to-peer learning and support amongst participants who might not otherwise communicate due to tribe divisions and gender segregation in Dadaab. The following WhatsApp message summarised this concept:

We are all one family (UNIGE-programme). Me and (student’s name) we are all here (location 1) and (location b). (7)

b) Application of their ICT skills beyond the course

Furthermore, students reported that their newly acquired ICT skills enabled them to not only access education but also job markets. They reported using online platforms for job searches in Somalia and Dadaab. While participants were very enthusiastic about technology, their preferred learning materials were still books. This finding prompted questions on course design, such as how key features of mobile devices could be exploited for educational purposes and how technology could be more harmoniously utilised alongside traditional materials such as books. This observation was particularly pertinent considering students had 4 h of electricity per day.

Education programmes and innovative ICT solutions also enhanced professional pathways for students in Dadaab. The following section presents information regarding what is available and how blended learning could open new opportunities for junior health professionals.

iv) Professional pathways available to refugee students

Training for junior health staff tends to be vocational in nature. It comprises short, targeted modules and is non-credentialed and camp based. Despite the recent opening of professional health education in Dadaab, the available opportunities have largely failed to create professional pathways for refugee learners that responded to the uncertainty and limitations of their realities.

- Education journeys

Most participants attended various health-related vocational courses, most of which were not credentialled and therefore had no value for professional development. Participants concurred that non-credential training was of limited value and students agreed that they would benefit from credentialed education, which could contribute to building professional pathways. Students’ application and recounts of their education journeys over the interviews show that students were used to arbitrarily moving from one training course to the next. Accessing education was celebrated amongst all participants, but building a career was not a strong consideration, perhaps because of a lack of options. The following quote represents a regular student having sampled many fields, before becoming interested in professional health education;

I used to be a teacher, then I did livelihoods but my friends told me to get into medical so I could get a job. (2)

b) Non-credentialed training

Managers perceived non-credential training as a hindrance. They believed that non-credentialed training stopped them from advocating for the promotion of their staff, as they would lack official documentation to sustain their training. Alongside legal constraints for refugees in Dadaab, this also prevented managers from promoting unskilled incentive staff to skilled incentive staff:

To be moved to skilled they need to have a diploma and licence, but refugees cannot get either....without diploma and licence is difficult. Now, (student) gained her certificate so she can be hired as skilled… manager could request a promotion but is up to the organisation to give the promotion, and ultimately UNHCR. (C)

Given that refugees in Kenya do not have the right to paid work, refugees can only participate in the workforce as incentive workers at the camp or seek employment in the informal sector. Incentives may include cash, vouchers or in-kind goods as compensation for work or services. Nowadays, there is no formal guidance on incentive work in Dadaab or other camps. It is difficult to say whether Kenyan law and policies could evolve to allow professionally trained health care workers to obtain formal jobs. Therefore, focusing on internationally credentialled courses and transferable skills would be a sensible approach for higher education participants.

c) Building professional pathways

While opportunities are limited in Dadaab, the Somali health workforce is characterised by serious shortages at all levels. It could offer students recognition of their skills, competitive salaries, and professional development. To meet the needs of students in Dadaab, professional health education enables synergies across relevant bodies and frameworks. Nonetheless, Somalia is not safe. Student views are divided; they fear that tribalism could hamper their opportunities or even put their lives at risk, but they are also optimistic, driven by a moral desire to rebuild their homeland. The following quotes illustrate these thoughts:

The majority clans are getting the jobs. Minorities will not get jobs even with degrees. (FG)

I will get a job in Somalia, Somalia is my country, if I go to USA or Geneva I am still Somali, I want to come back to Somalia. (FG)

Building professional pathways requires enhanced coordination across relevant bodies including states, international organisations, NGOs, and universities. Youth in Dadaab not only need access to professional health education, but they need access to professional pathways. The data show that 5 months after the completion of the course, there is a raising number of the graduates that are now being hired in junior posts in the health sector, both in Dadaab and in Somalia which is a positive and encouraging effect of this training. Health workers are at the heart of health systems, and enhancing the synergy between eligible young people, professional health education and the health system is essential.

## Discussion

Finding solutions for refugees requires addressing key issues regarding health professional education, refugee youth, migration and building capacity across the health workforce. This paper has outlined critical success factors for the implementation, design and adoption of e-Learning for junior health care workers in refugee camps. This research outlines several knowledge gaps. It suggests that blended learning enables the design and delivery of high-quality medical education that is sustainable and relevant in an environment, e.g. refugee camps. Furthermore, the research reveals that building education pathways could generate enough numbers of health workers with the appropriate skillset to serve communities. The country of origin and countries of resettlement are critical in achieving the global strategy on human resources for health. This global strategy calls for the transformation of health workforce education to support universal health coverage. It calls on states to develop partnerships between members, international organisations and non-state bodies such as educational institutions.

## Conclusion

As conflict continues to rise, more must be done to strengthen and update the tools and methods available to facilitate training of the healthcare workforce, as well as, cross-country accreditation mechanisms for health training institutions. Data from this study has shown that credited blended learning programmes are an efficient and strategic tool to strengthen junior health workforce on fragile contexts such as refugee camps. This type of training enhances synergies between eligible young people, professional health education local and international.

## Supplementary information

**Additional file 1. **a) Academic performance of enrolled students. b) Results of comparison of the overall scores of two groups : students with practical experience (group1) and students with no experience (group2). Comparison of the oral scores of two groups : students with practical experience (group 1)and students with no experience & Comparison of the scores of two groups : women (group 1) and men (group 2).

## Data Availability

This study involved qualitative data from a small sample of a vulnerable population. These data cannot be made publically available given the potential risk of identification. Special request for data access would be assessed alongside the Ethics Commission of the University of Geneva.

## Footnotes

1 In Kenya, refugees do not have the right to work. ‘Incentive work’ allows them to earn an income in the form of incentives as opposed to salaries. Incentives may include cash, vouchers or in-kind goods as compensation for work or services [24].

2 Refugee participants have been assigned a number or ‘FG’. It is used for quotes from focus groups. A letter is assigned to managers. Given the small number of participants, no gender or any other types of personal information will be referred to.

## Abbreviations

UNHCR: The United Nations High Commissioner for Refugees
NGO: Non-governmental organisation
UN: United Nations
MOOCs: Massive open online courses
BHER: Borderless Higher Education for Refugees Project
USB: Universal Serial Bus
OTG-B: USB On-The-Go adaptors
UNIGE: The University of Geneva
ICT: Information and communications technology
