# Supplementary material for: Critical success factors for the implementation and adoption of e-learning for junior health care workers in Dadaab refugee camp Kenya
Source: Hum Resour Health. 2019 Dec 9;17:98. doi: 10.1186/s12960-019-0435-8 (PMC6902417; doi:10.1186/s12960-019-0435-8)
Supplement: Supplementary file 1 — Additional file 1. a) Academic performance of enrolled students. b) Results of comparison of the overall scores of two groups : students with practical experience (group1) and students with no experience (group2). Comparison of the oral scores of two groups : students with practical experience (group 1)and students with no experience & Comparison of the scores of two groups : women (group 1) and men (group 2). [file 12960_2019_435_MOESM1_ESM.pdf]

Additional files

a) Academic performance

Results cohort 1

Student	Exa 1	Exa 2	Total written	Oral	Overall	Diploma
S01	37%	42%	39%	77%	58%	attestation
S02	50%	31%	42%			
S03	30%	50%	38%	42%	40%	attestation
S04	41%	58%	48%	38%	43%	attestation
S05	15%	50%	29%	45%	37%	attestation
S06	20%	39%	28%	52%	40%	attestation
S07	37%	39%	38%	52%	45%	attestation
S08	24%	33%	28%	47%	37%	attestation
S09	11%	75%	37%	38%	38%	attestation
S10	9%	42%	22%	62%	42%	attestation
S11	41%					
S12	11%	8%	10%			
S13	0%	0%	0%	30%	15%	certificate
S14	7%	8%	8%	39%	24%	certificate
S15						
S16						
S17						
**Total**	**24%**	**37%**	**28%**	**47%**	**38%**	

Results cohort 2

Student	Exa 1	Exa 2	Total written	Oral	Overall	Diploma
S21	56%	56%	56%	79%	67%	attestation
S22	48%	31%	41%	50%	46%	attestation
S23	7%	6%	7%	43%	25%	certificate
S24	17%	28%	21%	42%	31%	certificate
S25	39%					
S26	54%	47%	51%			
S27	28%	25%	27%	53%	40%	attestation
S28	43%	56%	48%	93%	70%	attestation
S29	41%	19%	32%	40%	36%	attestation
**Total**	**37%**	**34%**	**35%**	**57%**	**45%**	

B) Results of profile correlation

Comparison of the overall scores of two groups: students with practical experience (group1) and students with no experience (group2)

Méthode : Welch Two Sample t-test; Alternative :two.sided
Qobs : 1.0036413411336
p-value : 0.33667551242336
T : Array IC at 95%[-7.9724 ; 21.401]
Degree of liberty: 11.236449805607
Mean: Group 1: 47.857142857143 ; Group 2: 41.142857142857

Comparison of the oral scores of two groups: students with practical experience (group 1) and students with no experience (group2)

Method : Welch Two Sample t-test; Alternative :two.sided
Qobs : 0.97712770648394
p-value : 0.34944814333351
T : Array Intervalle de confiance à 95%[-11.4444 ; 29.7301]
Degree of liberty : 11.031214534765
Mean : Group 1: 58.428571428571 ; Group2: 49.285714285714

Comparison of two groups: with motivation towards clinical practice (group 1) and without (group 2)

Méthode : Welch Two Sample t-test; Alternative :two.sided
Qobs : 2.0081220225098
p-value : 0.10321537714509
T : Array IC at 95%[-4.3745 ; 33.9301]
Degree of liberty : 4.8033199924694
Mean: Group 1: 54 ; Group 2: 39.222222222222

Comparison of the scores of two groups : women (group 1) and men (group 2)

Method : Welch Two Sample t-test; Alternative :two.sided
Qobs : -0.85265805271521
p-value : 0.40745194036
T : Array Intervalle de confiance à 95%[-16.3831 ; 7.0293]
Degree of liberty : 14.782743122537
Mean : Group 1 : 37.4 Group 2 : 42

Comparison of two groups : without content on cell phone (group 1) and with cell phone (group 2)

Methode : Welch Two Sample t-test; Alternative
:two.side Qobs : -
0.97810157690588 p
-value :
0.34747179058998
T : Array Intervalle de confiance à 95%[-20.7222 ; 7.8888]
Degree of liberty:
11.911524536676
Mean: Group 1: 40.833333333333 ; Group 2: 47.25
